# Plant Genotype Shapes the Bacterial Microbiome of Fruits, Leaves, and Soil in Olive Plants

**DOI:** 10.3390/plants11050613

**Published:** 2022-02-24

**Authors:** Antonino Malacrinò, Saveria Mosca, Maria Giulia Li Destri Nicosia, Giovanni E. Agosteo, Leonardo Schena

**Affiliations:** Dipartimento di AGRARIA, Università Mediterranea di Reggio Calabria, 89122 Reggio Calabria, Italy; saveria.mosca@unirc.it (S.M.); giulia.lidestri@unirc.it (M.G.L.D.N.); geagosteo@unirc.it (G.E.A.); lschena@unirc.it (L.S.)

**Keywords:** microbiota, metabarcoding, 16S, *Olea europaea*

## Abstract

The plant microbiome plays an important role in plant biology, ecology, and evolution. While recent technological developments enabled the characterization of plant-associated microbiota, we still know little about the impact of different biotic and abiotic factors on the diversity and structures of these microbial communities. Here, we characterized the structure of bacterial microbiomes of fruits, leaves, and soil collected from two olive genotypes (Sinopolese and Ottobratica), testing the hypothesis that plant genotype would impact each compartment with a different magnitude. Results show that plant genotype differently influenced the diversity, structure, composition, and co-occurence network at each compartment (fruits, leaves, soil), with a stronger effect on fruits compared to leaves and soil. Thus, plant genotype seems to be an important factor in shaping the structure of plant microbiomes in our system, and can be further explored to gain functional insights leading to improvements in plant productivity, nutrition, and defenses.

## 1. Introduction

Plants associate with a plethora of microscopic organisms, including bacteria, archaea, eukaryotes and viruses, often designated as the plant microbiota. The structure of plant-associated microbial communities depends on a multitude of factors. For example, the abiotic environment has been reported to strongly drive the composition of the plant microbiome, including soil [1,2,3], atmosphere [4], geography [5,6], and many others. In addition, the plant microbiome is under the influence of many biotic factors, some of them exogenous like herbivory [1,7] or plant diseases [8,9,10,11,12,13,14,15,16,17], some others are instead driven by the plant itself. Indeed, previous studies reported variation in the structure of plant microbiomes according to compartment (e.g., root, leaf, fruit, flower) [18], but also within the same compartment, for example between different parts of flowers and fruits, or between internal and external tissues [18,19]. Plant genotype is a major driver of the structure of plant-associated microbial communities, an effect mainly driven by the different physical and chemical properties that characterize different compartments [18,19]. The effect of plant genotype in driving the structure of plant microbiomes has been reported in several systems, including *Boechera stricta* [20], *Medicago trunculata* [21], *Solanum tuberosum* [1], *Glycine max* [22], *Populus trichocarpa* [23], *Cucurbita pepo* [24], and many others, while in some other species plant genotype has been found to drive a weak signal, for example in *Triticum aestivum* [25], and *Phaseolus vulgaris* [26]. Thus, we are still not able to predict the strength of the effect driven by plant genotype on plant-associated microbiomes.

Predicting the effect of different factors on plant microbiomes can be pivotal in understanding the dynamics of these complex communities. Indeed, recent research is providing increasing evidence of the role of plant microbiomes in guaranteeing plant health [18]. In particular, it is important to understand how plant microbiomes vary according to plant genotype, as it might be key to understand the link between the plant genome and the plant microbiome, enabling microbiome manipulation to positively influence plant health. Among the many plant species, several studies targeted the microbiome of olive trees (*Olea europaea*), trying to understand the factors shaping their microbiome. For example, Fausto et al. [27] found that soil management impacted the microbial composition of leaves and xylem sap. Fernández-González et al. [28] found differences in the microbiome structure of root and rhizosphere. Other studies suggested differences between compartments. For example olive leaves, flowers and fruits show different fungal microbiomes [8]. Also the infection by *Xylella fastidiosa* has been found to influence the olive tree microbiome in different tissues, with an effect mostly dependent on the resistance to this pathogen [29,30]. In a study on 10 different olive genotypes, Müller et al. [31] found that the structure of communities of endophytes thriving in olive leaves reconciled with the plant origins in “Eastern” and “Western” areas of the Mediterranean basin. Similarly, the analysis of the foliar fungal endophytes in different varieties revealed a strong signature driven by plant genotype [32]. In a wider study on 36 genotypes cultivated under a common garden setup, Fernández-González et al. [28] found that plant genotype influenced the structure of root and rhizosphere bacterial and fungal communities. A signature of plant genotype was also detected when analyzing the xylem microbiota [33]. Although we still lack information about how different factors (e.g., plant genotype, abiotic/biotic stressors, agricultural practices) influence the olive microbiota at different compartments, and which is their relative strength, many studies support plant genotype as the major driver of microbiome structure in olive trees.

While several studies contributed to decipher the olive tree microbiome, and many supported a strong effect driven by plant genotype on both the bacterial and fungal communities [28,29,30,31,32,33], we still need to quantify the contribution of plant genotype in shaping the olive microbiota associated with different compartments. In this study, we contribute to fill this gap by characterizing the bacterial microbial communities associated with two different olive genotypes (Sinopolese and Ottobratica) at different compartments (fruits, leaves, and soil) and quantifying the strength of the effect driven by plant genotype at each compartment. Given that different plant genotypes strongly differ in the blend of VOCs (Volatile Organic Compounds) and exudates they release, we hypothesize that the effect driven by plant genotype would be stronger in plant-tissues (which are directly influenced by the plant) than soil.

## 2. Methods

### 2.1. Sampling

Samples were collected in an olive orchard located in the Gioia Tauro plain (Calabria, Italy) during October 2019. Within an area of ∼5 ha, we located 30 plants of two different olive varieties: Ottobratica and Sinopolese (*n* = 15 each). These are two local olive varieties mostly used to produce oil, and their phenology is pretty similar with full ripening of fruits around the end of October. This field was selected because it was cultivated with both varieties, and the climatic and pedological characteristics were homogeneous throughout the sampled area. From each plant we collected ∼10 leaves from different parts of the canopy, ∼10 fruits with the same strategy, and soil from 5 different points directly below the canopy, discarding the litter and not sampling beyond 10 cm from the surface. All collected leaves and fruit were apparently healthy as they did not show any symptom of disease, and fruits were ripe and ready to be harvested. Samples were temporarily stored at 4 °C for a few hours, and then at −20 °C until further processing.

### 2.2. DNA Extraction, Library Preparation, and Sequencing

Samples were pre-processed differently according to their source (soil, leaves, fruits). Soil samples (∼25 mg) were mixed with 300 µL of extraction buffer (10 mM Tris, 100 mM NaCl, 10 mM EDTA, and 0.5% sodium dodecyl sulfate) and crushed using three 1-mm-diameter stainless steel beads per tube with the aid of a TissueLyzer II (Qiagen) bead mill homogenizer set at 30 Hz for 5 min. Leaf samples were finely cut into small pieces using sterile scissors, freeze dried for ∼72 h, powdered with mortar and pestel using liquid nitrogen, and ∼25 mg were processed as soil samples above. Similarly, fruits were peeled, and the peer was cut into small pieces, freeze dried for ∼72 h, powdered and ∼25 mg were processed as above.

DNA was extracted from each sample using phenol/chloroform, then quantified and quality-checked using a Nanodrop 2000 spectrophotometer (Thermo Fisher). Metabarcoding analyses targeted the bacterial 16S rRNA gene using the primer pair 515f/806rB [34]. PCRs were performed in a 25 µL mix (∼50 ng of DNA, 0.5 µM each primer, 1 X KAPA Biosystems HiFi HotStart ReadyMix) using a Mastercycler Ep Gradient S (Eppendorf) set at 95 °C for 3 min; 98 °C for 30 s, 55 °C for 30 s, and 72 °C for 30 s repeated 35 times; and ending with 10 min of extension at 72 °C. A non-template control, replacing the target DNA with nuclease-free water, was included in all PCR assays. Libraries were checked on agarose gel for successful amplification and purified with an Agencourt AMPure XP kit (Beckman and Coulter) using the manufacturer’s instructions. A second short-run PCR was performed in order to ligate the Illumina i7 and i5 barcodes and adaptors following the supplier’s protocol, and amplicons were purified again as above. Libraries were then quantified using a Qubit fluorometer (Thermo Fisher Scientific), pooled together at equimolar ratio, and sequenced on an Illumina MiSeq platform using the MiSeq Reagent Kit v3 300PE following the supplier’s protocol.

Paired-end reads were processed using the DADA2 v1.22 [35] pipeline implemented in R v4.1.2 [36] to remove low-quality data, identify ASVs (Amplicon Sequence Variants) and remove chimeras. Taxonomy was assigned using SILVA v138 [37]. Reads identified as chloroplasts were removed from the downstream analyses.

### 2.3. Data Analysis

Data was analyzed using R v4.1.2 [36] with the packages *phyloseq* [38], *vegan* [39], *DESeq2* [40], and *lme4* [41]. We tested the influence of plant genotype on the diversity and structure of the bacterial microbiome at each compartment. The diversity of microbial communities was estimated for each sample using Faith’s phylogenetic diversity index [42]. We selected this index because it considers the phylogenetic relationship between the different components of the microbiome. Tests were performed by fitting a linear model specifying compartment (i.e., soil, leaf, and fruits), plant genotype, and their interactions as fixed factors. Models were fit using the *lm()* function and the package *emmeans* was used to infer pairwise contrasts (corrected using false discovery rate, FDR). Similarly, we tested the influence of plant genotype on the structure of bacterial microbiomes in our system using a multivariate approach. Data was normalized using *DESeq2* [40], and distances between pairs of samples, in terms of community composition, were calculated using a unweighted Unifrac matrix, then visualized using a canonical analysis of principal coordinates (CAP) procedure. Differences between sample groups were inferred through permutational multivariate analysis of variance (PERMANOVA) (999 permutations), specifying compartment, plant genotype, and their interactions as fixed factors.

For each compartment, we also tested which ASVs varied in relative abundance as response to plant genotype. Using *DESeq2*, we built a model for each compartment including plant genotype as fixed factor, extracting the appropriate contrasts (Sinopolese vs. Ottobratica), and filtering ASVs with a FDR-corrected *p* < 0.05. We also attempted to quantify the impact of plant genotype on influencing the bacterial microbiome at each compartment using two methods. First, we tested the impact of plant genotype separately on each compartment using PERMANOVA. Second, using *DESeq2*, we calculated the effect of plant genotype on the abundance of each ASV (expressed as absolute log${}_{2}$ fold changes) at each plant compartment. To do so, we built a model for each compartment using plant genotype as fixed factor, and then we extracted the appropriate contrasts (Sinopolese vs. Ottobratica). From each contrast, we used the absolute log${}_{2}$ fold-change values for each ASV to quantify the impact of plant genotype on the microbiota in each compartment. Comparisons of absolute log${}_{2}$ fold-change values were performed by fitting a linear mixed-effects model, specifying compartment as fixed factor and ASV identity as a random effect, and using the package *emmeans* to infer contrasts (FDR corrected).

The network analysis was performed using the R package NetCoMi [43], testing differences in network metrics between pairs of network using the function *netCompare()*.

## 3. Results

### 3.1. Description of Bacterial Communities

**Figure 1:**
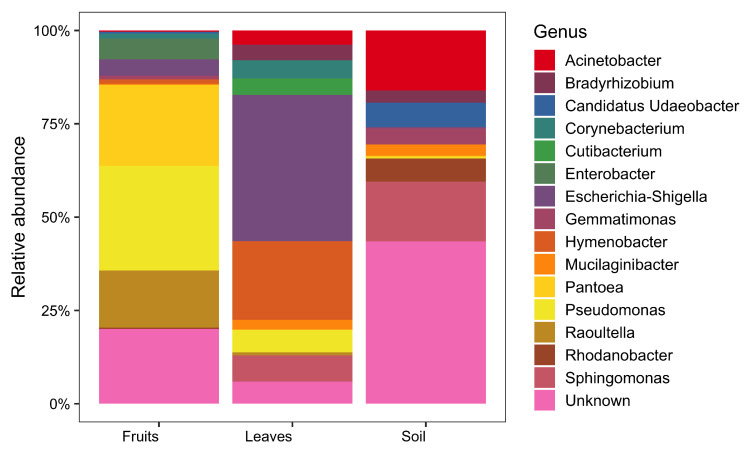
Bacterial community composition at each compartment (fruits, leaves, soil, combined for both genotypes). Bacterial genera with a relative abundance <1% are not reported.

Metabarcoding analyses identified 2498 different ASVs in our system (Figure 1). In soil samples, we mostly found unidentified taxa (43.97%), *Acinetobacter* (16.06%), and *Sphingomonas* (16.04%), while in leaves we found a higher amount of unidentified *Escherichia-Shigella* (39.17%) and *Hymenobacter* (21.05%). The fruit microbiome was dominated by *Pseudomonas* (28.05%), *Pantoea* (21.66%), and unknown taxa (40.02%).

### 3.2. Plant Genotype Influences Diversity and Structure of Olive-Associated Bacterial Microbiota

**Figure 2:**
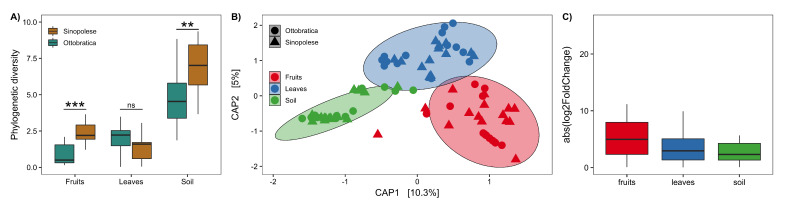
(**A**) Comparison of Faith’s phylogenetic diversity index between plant genotypes (Sinopolese, Ottobratica) across compartments (fruits, leaves, soil). *** *p* < 0.001, ** *p* < 0.01, ns = *p* > 0.05. (**B**) Canonical analysis of principal (CAP) coordinates ordination using a Unifrac distance matrix of samples. Percentages in parentheses report the variance explained by the respective axis. (**C**) Magnitude of changes in abundance for each ASV (absolute log2 fold changes). For each compartment (fruits, leaves, soil), we investigated the response of single ASVs to the plant genotype.

Both phylogenetic diversity and microbiota structure were influenced by the interaction between compartment × plant genotype (Table 1, Figure 2). Post-hoc contrasts on phylogenetic diversity analysis revealed that soil (*p* < 0.0001) and fruits (*p* = 0.0014) microbiome diversity was influenced by plant genotype, with higher values in samples coming from the Sinopolese genotype, while leaves (*p* = 0.2919) microbiome diversity was not affected by this factor (Figure 2A). Instead, post-hoc contrasts on the multivariate model show differences between plant genotypes in soil (*p* = 0.006), leaves (*p* = 0.039), and fruits (*p* = 0.001).

### 3.3. Plant Genotype Impacts Fruits Bacterial Microbiota More Than Leaves or Soil

We also attempted to quantify the impact of plant genotype on the structure of microbiota at each compartment using two different approaches. First, we run a PERMANOVA separately for each compartment, and we found that plant genotype explained 22.96% of the variation in fruits (F = 8.34, *p* < 0.001), 5.70% on leaves (F = 1.63, *p* = 0.06), and 6.45% on soil (F = 1.86, *p* = 0.003). Second, we tested the impact of plant genotype in shifting the relative abundance of each ASV, and used it as a metric to estimate the shift in microbiome composition. While fruits showed higher absolute log${}_{2}$ fold-change values than leaves and soil (Figure 2C), the linear mixed-effect model did not suggest any difference between these groups ($\chi^{2}$ = 4.75, df = 2, *p* = 0.09).

We then tested which bacterial taxa were influenced by plant genotype at each compartment. In fruits, we found 96 differentially abundant ASVs, of which 38 *Pseudomonas* and 8 *Escherichia-Shigella* were more abundant in samples from the Sinopolese genotype, while 39 *Raoultella*, 1 *Klebsiella*, and 10 unknwon were more abundant in fruits from Ottobratica. In leaves, we found only 12 *Hymenobacter* ASVs that were more abundant in samples from Ottobratica plants. In soil, we found 4 *Sphingomonas* ASVs that were more abundant in samples collected below the canopy of Sinopolese plants, while 1 unidentified ASV was more abundant in soils collected in proximity of Ottobratica plants.

Finally, we tested whether plant genotype impacts the bacterial co-occurence network within each compartment. In fruits we found differences in the degree (*p* < 0.001), closeness centrality (*p* < 0.001), and hub taxa (*p* = 0.027) as response to plant genotype. On the other hand, we did not find any impact of plant genotype on the co-occurence network within leaves and soil compartments (*p* > 0.05).

## 4. Discussion

In this study, we characterized the bacterial microbiota associated with fruits and leaves of two olive tree genotypes (Sinopolese and Ottobratica), together with the bacterial microbiota of the soil collected below the tree canopy. Our results show that microbiome composition mainly cluster by compartment (fruits, leaves, soil), but also plant genotype influenced its diversity and composition, in a way that mostly depended on the compartment × genotype interaction.

Previous studies tested whether olive plant genotype would influence the microbiome structure in different plant compartments. For example, Giampetruzzi et al. [29] studied the xylem microbiota of two olive genotypes (Kalamata and FS17) and compared its response to the infection by *X. fastidiosa*. While they found a different microbiota according to the infection status, they did not identify differences driven by plant genotype. Similarly, another study tested a similar question on a different pair of varieties (Cellina di Nardò and Leccino) focusing on both the xylem and leaf microbiomes, and found no differences in the structure of microbial communities between the two genotypes when they were not infected by *X. fastidiosa*, while they found a genotype-driven effect when plants were infected by the pathogen [30]. On the other hand, the analysis of the phyllosphere endophyte fungal communities of five different cultivars (Cobrançosa, Galega vulgar, Madural, Picual, Verdeal Transmontana) found a strong signal driven by plant genotype [32]. A wider study on ten olive genotypes collected in areas across the mediterranean basin, revealed that the phyllosphere endophytes clustered into two main groups (“Eastern” and “Western") according to the origin of each variety [31]. A strong effect driven by plant genotype was detected in the olive tree root and rhizosphere microbiomes from a common garden experiment that included 36 olive varieties [28]. Our results agree with several of these examples [28,31,32], while contrast with others [29,30]. This might be because the two studies our results disagree with [29,30] focused on the olive xylem, while the others focused either on the phyllosphere or belowground tissues. Thus, the effect driven by plant genotype might be weaker in internal tissues, like xylem, compared to other compartments more directly exposed to the environment.

We also found that plant genotype explained a higher proportion of the microbiome variation in fruits (∼23%), than leaves and soil (∼6%). Similarly, we found a higher number of ASVs that are differentially abundant between plant genotypes in fruits (*n* = 96), than leaves (*n* = 12), and soil (*n* = 5). Also, the network analysis suggests the impact of plant genotype on the microbial co-occurence network in fruits, while this was not observed for leaves and soil. Taken together, these results suggest that plant genotype has an influence on several plant compartments, including the soil below the tree canopy, but this effect is stronger in fruits than other compartments. To the best of our knowledge, only a previous study found a strong genotype-driven effect on the root and rhizosphere of olive trees [28]. This is not surprising, as previous studies found that plants influence the microbiome of each compartment through the release of VOCs and exudates, and this might be species and genotype dependent [18]. In our case, the microbiome of leaves and fruits might have been influenced by the genotype-specific chemical characteristics and by the production of genotype-specific VOCs [44]. On soil, these effects might have been driven by differences in the production of exudates and VOCs by the root system of the two genotypes [45,46], but also by different chemicals released by the degradation of leaves of each plant genotype [47]. The relative strength of plant genotype on several plant compartments has been tackled by few studies, for example our previous study on *S. tuberosum* [1]. The magnitude of the genotype-driven effect seems to vary according to plant species and compartment within each plant, although it is still hard to predict. In our case, the plant genotype-driven effect was detected at all compartments, but it was stronger in fruits than leaves and soil. The higher magnitude on fruits than leaves might be explained by the fact that samples were collected towards the end of the plant annual cycle, and probably the effect of plant genotype on leaves was masked by the effect of the environment (which is homogeneous between plant genotypes). On fruits the genotype-driven effect was still detectable because they were just reaching maturity and the two genotypes slightly differ in fruit morphological and chemical characteristics, and in the timing of fruit development.

Our results also suggest a high proportion of *Pseudomonas* and *Pantoea* in olive fruits, with several ASVs identified as *Pseudomonas*, *Escherichia-Shigella*, *Raoultella*, and *Klebsiella* being responsive to the olive genotype. Members of the genus *Pseudomonas* are widely known to be associated with a variety of plants, playing different roles including being endophytes, pathogens, or beneficial. Similarly, strains of *Pantoea* have been reported as both pathogens [48] and beneficial [49]. The other bacterial genera *Escherichia-Shigella*, *Raoultella*, and *Klebsiella*, have not been previously reported to play a major role within the fruit microbiome. Our results also suggest that *Hymenobacter* is a major component of the microbiome of olive leaves, and several ASVs have been found to shift between varieties. This genus has been previously found to be associated with the olive phyllosphere [27,33], but its functional role is still undisclosed. Soils were mostly characterized by unidentified ASVs, which is not surprising since the microbiome of soil is not well described, especially because we are still not able to cultivate in vitro the majority of soil microbial diversity.

Taken together, our findings suggest that plant genotype has a deep influence on the plant microbiome, with potential effect on the plant-microbe relationship. A deeper study to understand the mechanisms behind this effect might enable the manipulation of the plant microbiome to improve plant nutrition, fitness, and defenses. Also, this genotype-driven effect was observed in the microbiome of soil collected below the tree canopy. This effect is worthy of further investigation, as changes in the soil microbiome might result in consequences on the entire system, as soil microbiome is responsible for several functions including nutrient cycling. We are now fully aware of the powerful influence the microbiome has on its host plants, and we are now learning how to manipulate and exploit its function to improve plant productivity, quality, and protection, with positive impact on food security and safety, or the protection of natural resources.

## Figures and Tables

**Table 1:** Results from testing the effect of compartment (soil, leaves, fruits), plant genotype (Sinopolese, Ottobratica), and their interaction on the structure (PERMANOVA) and phylogenetic diversity of bacterial microbiota.

		PERMANOVA			Phylogenetic Diversity	
	df	R${}^{2}$	F	*p*	F	*p*
Compartment	2	0.15	8.42	<0.001	92.58	<0.001
Plant genotype	1	0.03	3.34	<0.001	15.49	<0.001
Compartment × Plant genotype	2	0.07	3.87	<0.001	8.64	<0.001

## Data Availability

Raw data is available at NCBI SRA under the bioproject PRJNA809643. The code used to analyze the dataset is available at https://github.com/amalacrino/olive_16S_microbiota, accessed on 17 February 2022.
